# Rogueing or Rescuing? A Potential New Management Approach for Roses Infected with Rose Rosette Virus

**DOI:** 10.3390/v17060829

**Published:** 2025-06-08

**Authors:** Caleb Paslay, Akhtar Ali

**Affiliations:** Department of Biological Science, The University of Tulsa, Tulsa, OK 74104, USA; cap1050@utulsa.edu

**Keywords:** roses, rose rosette disease (RRD), rose rosette virus (RRV), negative strand, segmented RNA virus

## Abstract

Roses (*Rosa* spp.) are among the most economically and culturally significant flowering plants worldwide. However, rose cultivation faces a critical threat from rose rosette disease (RRD), which is caused by *Emaravirus rosae* (rose rosette virus, RRV), a negative-sense RNA virus transmitted by the eriophyid mite *Phyllocoptes fructiphilus*. Current RRD management strategies mainly depend on the complete removal (rogueing) of symptomatic plants, which are effective but adds high economic and aesthetic costs. During our field and laboratory observations from 2023 to 2024, we documented that RRV often remains localized to a single cane for extended periods of time (up to 80 days) in one variety before systemic spread to other canes of the same plant. This discovery supports a proposed “rescue hypothesis”, suggesting that early pruning of symptomatic canes may prevent full-plant infection and serve as a viable alternative to rogueing under specific conditions. While preliminary, our findings offer a potentially cost-effective, less destructive management strategy. However, further research is needed to validate this hypothesis and inform integrated disease management practices are established for effective control of RRD.

## 1. Introduction to Roses

Roses (family: *Rosaceae*, subfamily: *Rosoideae*, genus: *Rosa*) are among the most economically significant and widely cultivated flowering plants worldwide. Roses are valued not only for their symbolic association with love, friendship, and beauty, but also for their diverse industrial applications. In 2022, the global cut flower industry was valued at approximately $9.66 billion globally, with rose accounting for an estimated 32% of this total value [1].

The United States (U.S.) was the largest importer of roses in that year, with imported values between $713 million and 800 million, while exports totaled approximately $2.9 million according to data from the OEC (Observatory of Economic Complexity). Beyond their ornamental appeal, roses are also important for their biochemical and antimicrobial contents [2] which contribute to their use across multiple sectors, including food and beverage, cosmetics, and aromatherapy. Furthermore, roses are widely utilized in landscape designs due to their aesthetic qualities and morphological diversity. Despite their value and versatility, roses are vulnerable to several diseases, among which rose rosette disease (RRD) is one of the most prominent and well established.

## 2. Rose Rosette Disease and the Negative Strand RNA Virus

Rose rosette disease (RRD) has been observed in rose plants since the 1940s, with its first description based on symptomatic observations published in 1941 [3]. For approximately seven decades thereafter, the identity of the etiological agent responsible for RRD remained elusive, despite the disease’s increasingly detrimental impact on rose cultivation throughout North America. Public gardens, botanical collections, and urban landscapes across the United States have experienced widespread losses due to RRD. As a result, growers and commercial landscapers have exhibited growing reluctance to cultivate roses, citing concerns over the disease (personal communication).

**Figure 1:**
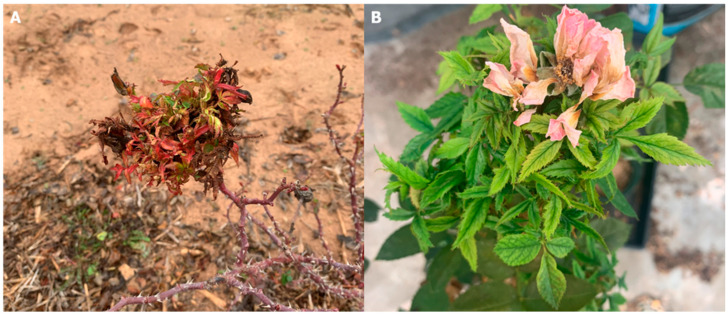
Images of roses that are (**A**) infected with RRV showing the common rosette or bunchy appearance (witches’ broom) and (**B**) leaf deformation and vein banding.

Historically, diagnosis of RRD relied primarily on characteristic symptoms, including rosette formation, persistent reddening of foliage, and excessive thorn proliferation (Figure 1). Transmission of the disease was linked to the eriophyid mite *Phyllocoptes fructiphilus* [4], supporting the hypothesis of a viral etiology. Subsequent research demonstrated that *P. fructiphilus* frequently associates with floral tissues, including sepals and petals [5].

In the early 2010s, *Emaravirus rosae*, commonly referred to as rose rosette virus (RRV), was conclusively identified as the causal agent of RRD [6]. RRV possesses a segmented, negative-sense RNA genome consisting of seven segments (RNA1–RNA7). Recent research on RRV has focused on several fronts, including the development of molecular diagnostic tools [7], breeding for host plant resistance [8], creation of infectious clones for reverse genetic studies [9], and investigations into the virus’s population structure [10].

## 3. Current Management Practices

The current literature identifies the primary management strategy for RRD as the removal of infected roses, including their roots and soil, a method hereafter referred to as the rogueing hypothesis [11,12]. It is highly recommended that the infected plant be removed for successful management of the disease [13]. Consequently, rose gardens affected by RRD have often been abolished entirely, leading to significant negative impacts on landscape aesthetics due to the absence of roses. While the rogueing hypothesis is a logical approach, it imposes substantial costs on private growers and the commercial rose industry, as it requires replanting and the redistribution of many roses. Additionally, the loss of entire plants can also discourage growers from continuing to invest in rose cultivation.

Although RRV is primarily confined to North America, major rose producing countries like the Netherlands and Ecuador would face severe consequences if outbreaks were to occur. In such scenarios, large-scale quarantines and plant removals would likely be necessary, posing serious risks to both local and global economies. Should RRV be detected in these regions, reliance on the current management approach (rogueing hypothesis) could prove devastating for commercial rose production.

## 4. General Observations

Over the past three years, our research has focused on the management of RRV and identification of resistance in a range of rose varieties. During our field visits to commercial rose farms and nurseries in 2023–2024, we had direct interactions with landscapers and gardeners and observed RRV infected plants. We witnessed widespread concern within the gardening and landscaping communities. RRV is commonly regarded as “the malady of roses”, and the fear surrounding it is significant.

In one such instance, we pointed out RRD-like symptoms on a single cane of a White Knock Out^®^ rose to a nursery worker. Without hesitation, he discarded the entire plant and no alternative management options were considered. This is especially consequential given that a typical rose plant costs between $20–25 and requires 6–18 months from initial propagation to be market-ready, depending on the desired size.

A similar approach was observed at the Tulsa Rose Gardens in Tulsa, OK, where the same rogueing strategy was applied regardless of disease severity or the number of canes affected. This practice of removing whole plants, even when only a single cane is symptomatic, is widespread and carries substantial economic implications for the rose industry. In 2024 alone, we documented RRD-like symptoms on more than 15 rose plants collected from various nurseries. In each case, symptoms were limited to a single cane, with no visible signs of infection on the remaining canes.

Previous studies have shown that RRV can be present without symptoms in some cases [11,13]. While discarding the entire plant may seem prudent, especially given the risk of asymptomatic infections, it is important to note that the presence of RRD on one cane does not necessarily imply systemic infection of the entire plant.

## 5. Laboratory Observations

In a controlled laboratory setting, we observed that the spread of rose rosette disease (RRD) from one cane to another within the same plant occurs at a relatively slow rate. For instance, on 30 July 2024, we obtained an Orange Glow Knock Out^®^ rose from a local plant nursery exhibiting RRD-like symptoms confined to a single cane, which was confirmed RRV-positive via RT-PCR. The exact source of the infection was unknown, as the plant was already infected before we acquired it.

The plant was monitored daily, and leaf tissues from each cane were tested weekly using RT-PCR. Typical RRD symptoms only appeared on a second cane on 18 October 2024, approximately 80 days after the initial observation of symptom detection on the first cane. The emergence of RRD-like symptoms and RT-PCR confirmation of RRV on the second cane suggest a gradual spread of the virus within the plant. In another case, a White Knock Out^®^ rose was visually monitored on a weekly basis. More than 180 days passed between the initial appearance of symptoms on a single cane and their subsequent appearance on a second cane, indicating a potentially prolonged latency or gradual spread of infection. While the initial symptomatic cane was confirmed as infected via RT-PCR, subsequent monitoring relied solely on visual symptom observation. While the underlying mechanism warrants further investigation, these findings have significant implications for exploring and developing alternative management strategies that may allow for targeted removal or treatment, rather than the complete destruction of entire rose plants.

## 6. Rescue Window of Virus-Infected Roses

Building on our observations of RRV’s apparently slow systemic movement, along with prior reports from rosarians [14], we propose that it may be possible to rescue RRV-infected plants if the initially symptomatic cane is pruned early, an approach we refer to as the rescue hypothesis. At minimum, this targeted pruning could limit the systemic spread of the virus within the plant and reduce sources of inoculum available to the mite vector. At best, it may prevent further transmission to surrounding roses and potentially save the infected plant from full systemic infection.

While previous studies have established that RRV spreads systemically and caution against relying solely on pruning as a management strategy [14], the rate at which the virus spreads from one cane to another has not been thoroughly investigated. Our findings suggest that this rate may be slow enough to allow for intervention through timely pruning of the affected cane.

## 7. Tentative Recommendations for Rose Growers

Based on our field and laboratory observations, we propose that pruning the initially symptomatic cane within a window of 1 to 80 days may help prevent systemic infection, a strategy we refer to as the rescue hypothesis. However, we strongly recommend removing the diseased cane as soon as symptoms are recognized to minimize the risk of further spread to the remaining canes. This will likely reduce the overall virus load and mite vector populations. This recommendation applies specifically to cases where only a single cane is symptomatic.

**Figure 2:**
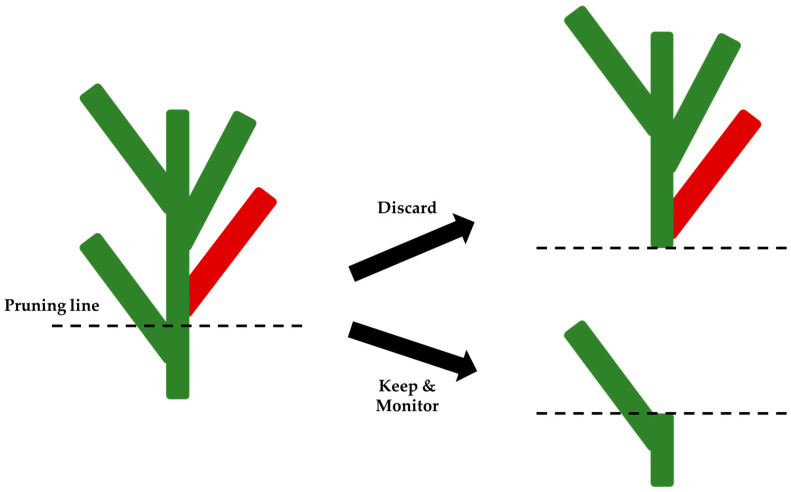
Illustration of RRV-infected rose plant showing the approximate area (in red) where the diseased portion may be pruned from the healthy remainder of the plant.

Although the specific pruning method has not yet been experimentally validated, we currently recommend pruning 8–10 inches below the infected cane, as shown in Figure 2. Based on current observations, RRV appears to spread upward through the plant. Therefore, removing tissue below the symptomatic region may be an effective strategy. It is also advisable to thoroughly clean pruning tools with soap, detergent, or a bleach solution before, between, and after use to minimize the risk of spreading the virus.

In situations where multiple canes exhibit RRD-like symptoms simultaneously (as reported in personal communications with other researchers), systemic infection is more probable, and complete removal of the plant (rogueing) is advised. Similarly, if a symptomatic cane remains on the plant for more than 80 days without removal, our data suggest that systemic spread is probable, and we again recommend discarding the entire plant.

Although pruning as a management strategy has been previously tested to a limited extent, promising results were reported: when pruning was conducted at the first sign of symptoms, 68% of rose bushes remained symptom-free one year later [15]. In contrast, delayed pruning led to symptoms in 72% of bushes within the same period. It is important to note, however, that these plants were not tested for RRV presence by RT-PCR or any other diagnostic assay, so asymptomatic infections may have gone undetected.

One advantage of pruning is the rose plant’s natural resilience; it can generate new, potentially uninfected canes and flush after removal of the diseased tissue. This makes pruning a potentially cost-effective and low-labor management option. Nevertheless, the context in which the disease occurs must be considered. The rate of systemic spread is likely influenced by multiple factors, including host genotype, viral load and accumulation, vector pressure, seasonal timing, and virus latency [13]. Until further research provides conclusive evidence to support or refute the rescue hypothesis, current best management practices should continue to be followed.

## 8. How Could This Change Our View of RRV?

It is well established that RRV poses a serious threat to rose cultivation and should not be underestimated. Nonetheless, we remain optimistic that alternative management strategies can be developed to ease the burden of RRV on homeowners, landscapers, gardeners, and plant nurseries. In cases of severe infection, complete removal and destruction of the affected plants may still be necessary. However, as discussed above, there may be a critical window during which infected plants can be rescued from systemic infection, a concept that requires further investigation.

One promising area of ongoing research is the development of RRV-resistant rose cultivars [8]. As efforts to breed resistant germplasm progress, pruning may serve as a valuable interim strategy. In the future, a combined approach using both pruning and resistant cultivars could significantly reduce the spread and impact of RRV, potentially bringing disease management to more sustainable levels.

## 9. Conclusions

It is important to emphasize that our observations have not yet been fully validated through systematic experimentation. However, they suggest a possible vulnerability in the virus’s capacity to spread systemically within the plant’s vascular system. We encourage fellow researchers to explore alternative, cost-effective RRD management strategies and to investigate the apparent delay in RRV’s systemic movement.

While we acknowledge the caution exercised when RRD symptoms are observed and continue to recommend adherence to current management practices, we propose that this new rescue hypothesis, in contrast to the traditional rogueing hypothesis, offers a potentially viable alternative. The rogueing hypothesis assumes inevitable systemic spread and advocates for full plant removal to minimize inoculum. Conversely, the rescue hypothesis postulates that early pruning of symptomatic canes may offer a less destructive and more practical solution.

Pending future experimental validation, we encourage rose growers to consider pruning visibly affected canes rather than removing entire plants, while continuing to follow existing guidelines. In doing so, we take a step closer to advancing RRV management and building on seven decades of research, from the initial description of the disease to a deeper understanding of its biology and control.
